# Targeted macrophage mannose receptor (CD206)-specific protein delivery via engineered extracellular vesicles

**DOI:** 10.1016/j.heliyon.2024.e40940

**Published:** 2024-12-06

**Authors:** Leyla A. Ovchinnikova, Daria Y. Tanygina, Samir S. Dzhelad, Evgeniy G. Evtushenko, Dmitriy V. Bagrov, Alexander G. Gabibov, Yakov A. Lomakin

**Affiliations:** a Shemyakin-Ovchinnikov Institute of Bioorganic Chemistry RAS, Moscow, Russia; b Faculty of Chemistry, Lomonosov Moscow State University, Moscow, Russia; c Faculty of Biology, Lomonosov Moscow State University, Moscow, Russia

## Abstract

Extracellular vesicles (EVs) show great potential for therapeutic delivery to human cells, with a focus on modulating immune responses. The most promising targets for inducing humoral and cellular immunity against a specific antigen are macrophages (Mϕs) and dendritic cells (DCs). Targeting mannose receptors (CD206), which are highly expressed on these antigen-presenting cells, to promote the presentation of specific antigens through EV-mediated uptake, is a promising strategy in clinical immunotherapy. Our study compares two EV-fused anti-CD206 nanobodies in delivering cargo proteins to human activated antigen-presenting cells. We demonstrated that nanobody-functionalized EVs exhibit enhanced interaction and increased uptake by CD206^+^ cells compared to non-targeted EVs. Furthermore, replacing the full-length vesicular stomatitis virus protein G (VSV-G) with its truncated form, fused to a monoclonal anti-CD206 nanobody, significantly improves the specificity of EV uptake by CD206^+^ cells. Our study outlines an optimized platform for the production of targeted EVs designed for specific protein delivery to CD206-positive human cells.

## 1 Introduction

The worldwide spread of various viral, cancer, and autoimmune diseases highlights the urgent need for an efficient platform capable of modulating the immune response in a controlled manner. Antigen-presenting cells (APCs) play a pivotal role in initiating the required T-cell stimulation, thereby contributing to the subsequent development of the immune response. Dendritic cells (DCs) and macrophages (Mϕs) are, in turn, the most effective APCs serving as key initiators of primary adaptive immune responses [1]. Therefore, targeted delivery of necessary antigens to these cells would enable the activation of protective T cells, leading to the suppression of viral and bacterial infections, or potentially controlling tumor growth. In the context of the autoimmune diseases, an enhanced presentation of self-antigens can induce immune tolerance of the autoreactive T cells and reduce autoinflammation [2].

Extracellular vesicles (EVs) are naturally occurring nanocarriers that are secreted by almost all cell types [3]. EVs are highly heterogeneous and can be classified into two main subtypes based on size, density, molecular composition, and biogenesis: microvesicles, or ectosomes (100–1000 nm), and exosomes (50–150 nm). In addition, apoptotic bodies (1–4 μm) are identified as the third, very specific subtype of EVs [[4], [5], [6]]. Since EVs are capable of transferring proteins, lipids, DNAs, and RNAs between cells, their role extends beyond intercellular signaling, and they are known to contribute to diverse immune responses [7] and cancer development [8,9]. EVs are known for their ability to serve as potential biomarkers for the diagnosis or monitoring the progression of various diseases [10]. At the same time, their biocompatibility [11] and ability to penetrate the brain-blood barrier [12], make them promising agents for drug delivery [13].

Genetically engineered EVs can be modified by displaying targeting molecules on their surface [[14], [15], [16], [17]] and loading them with the specific cargo [3,14,18,19]. Genetically boosted EVs have been shown to deliver specific mRNAs into the cytosol of target cells, representing a potential avenue for the treatment of neurodegenerative disorders [20]. A platform based on the interaction of CD63 fused with the aptamer-binding protein Com and com‐modified sgRNA was developed to actively enrich EVs with ribonucleoproteins (RNPs) such as Cas9 RNPs [21]. Another version of the EV-based RNPs delivery system was termed “gectosomes” [22]. In gectosomes, cargo loading is provided by co-encapsulation of the vesicular stomatitis virus G protein (VSV-G) with the delivered molecules through split GFP complementation. This mechanism enables the transfer of various gene-editing proteins, including Cre and SaCas9.

Meanwhile, the full potential of utilizing EVs as biomarkers or drug carriers is severely limited by the absence of a standardized and reliable technique for their isolation. Diverse strategies, such as size-based separation, density gradient centrifugation, and surface marker targeting, are employed for isolating and purifying EVs. Each approach has unique benefits and limitations [23]. Combining several methods of EV isolation has proven to be more efficient than a single approach [23,24]. Low-speed centrifugation, ultrafiltration, and size exclusion chromatography (SEC) represent a promising combination of methods for EV isolation from cell culture media [25], which remains the preferable source material [26].

Previously, we increased the loading capacity of engineered EVs by replacing their toxic component, Vpr, with a pair of leucine domains from Jun and Fos proteins utilized to package cargo molecules into EPN (enveloped protein nanocages) [14]. These specific EVs are generated by transfecting eukaryotic cells with the genetic constructs encoding the following proteins: VSV-G for EV budding from producer cells, EPN self-assembling domain allowing cargo loading into EVs, and the desired cargo protein itself [27]. In the present study, we modified the surface of EVs to achieve their targeted delivery to APCs and reduce nonspecific background binding. For this purpose, we utilized a truncated form of VSV-G protein fused with two variants of llama nanobodies (Nbs) targeting the mannose receptor (CD206), which is highly expressed on the surface of APCs [28,29]. Ultrafiltration and SEC were employed to obtain purified engineered EVs, thereby preserving their integrity and functionality.

## 2 Results

### 2.1 EV isolation and characterization

**Fig. 1:**
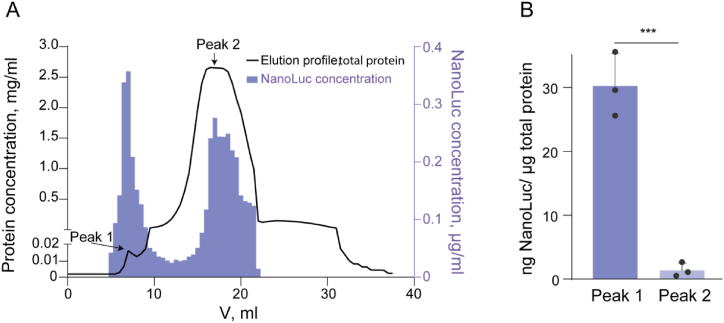
**EV purification by SEC**. (**A**) Fraction distribution of NanoLuc (violet) and total protein concentrations (black). Thirty-five 500 μl fractions were collected, and the presence of NanoLuc was determined in each fraction by the NanoGlo Assay. (**B**) The NanoLuc/total protein ratio of the eluted peaks, corresponding to the combined fractions 4–6 (peak 1) and 24–30 (peak 2). The data are presented as mean ± SD, n = 3. ∗∗∗p < 0.001, two-tailed Student's t-test.

At the outset, we optimized the protocol for producing and purifying engineered EVs designed to deliver specific cargo to target cells. We used pseudotyped EVs with active cargo loading into the self-assembling EPN nanocages [27], which in turn, were encapsulated into VSV-G-containing EVs by recruiting the endosomal sorting complexes required for transport (ESCRT) machinery. As a proof of concept, we investigated EVs loaded with recombinant luciferase NanoLuc. To reduce the content of natural EVs in conventional cell medium, we used exosome-depleted FBS for EVs production. After transfecting HEK293T cells, EVs isolated from medium were subjected to differential low-speed centrifugation followed by ultrafiltration. SEC was implemented to increase the purity of the engineered EVs by isolating them from the residual components of the collection medium. The EV concentrate was applied to the Superose 6 column, and 35 fractions (each containing 500 μL) were collected for each analysis. Two separate elution peaks were observed (Fig. 1A). According to the previously published data, the first peak corresponded to EVs, and the second peak was related to free soluble protein [30]. Although NanoLuc was detected at comparable levels in both elution peaks (Fig. 1A), the correlation of NanoLuc with total protein was 30 times greater for peak 1 than for peak 2 (Fig. 1B). Therefore, the SEC-purified engineered EVs described herein enable the loading of NanoLuc up to 3 % of the total protein content in the preparation.

**Fig. 2:**
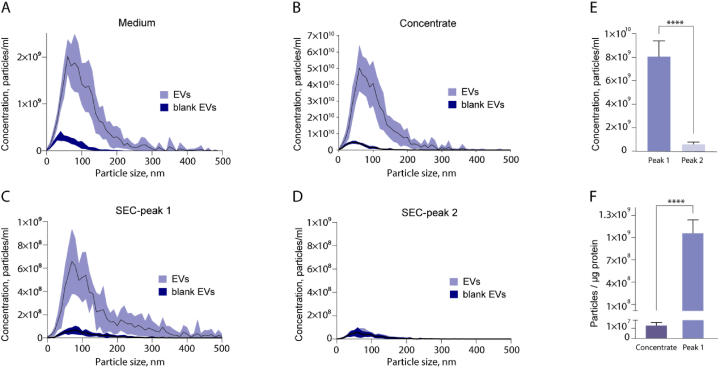
**The NTA analysis of engineered EVs.** (**A**–**D**) Size distribution of EVs isolated from HEK293T cell culture media estimated by NTA. The separated samples after differential low-speed centrifugation (**A**), ultrafiltration (**B**), and SEC (**C, D**) were analyzed. The graphs demonstrate the average number and size (solid line) of particles with SD (shaded area). (**E**) Particle concentrations of peaks 1 and 2. (**F**) The particles/total protein ratio of the samples. EVs – engineered EVs, blank EVs – collection media used as a negative control. ∗∗∗∗p < 0.0001, two-tailed Student's t-test**.**

To further characterize the engineered EVs, the samples obtained at the different stages of purification were analyzed by NTA (Fig. 2A–D). Previously, it was shown that adventitious nanoparticles found on EV preparations can reach up to one-third of the total EV amount [31]. To ensure that our investigation focused predominantly on the size of EVs rather than adventitious nanoparticles from the culture medium, plastic and SEC column, we performed additional NTA of the nonconditioned medium passed through all purification steps as a reference (blank EV). The particle size distribution in all samples was in the range of 30–300 nm, while the mean size was 108 ± 10 nm for the concentrated EVs and 152 ± 28 nm for the purified EVs from peak 1, which is typical for EVs. As expected, the particle concentration in the peak 1 (8.1 ± 1.3 × 10^9^ particles/ml) was significantly higher than that in peak 2 (0.6 ± 0.2 × 10^9^ particles/ml) (Fig. 2E). The portion of adventitious nanoparticles in the medium, concentrate and peak 1 ranged from 7 to 11 %. At the same time, the particle concentrations in peak 2 for both the purified EV sample and the purified blank EV sample were similar (0.5 ± 0.2 × 10^9^ particles/ml). Therefore, it can be concluded that no engineered EVs were detected in peak 2. To estimate the efficiency of eliminating extraneous proteins during EV purification, total protein amount was measured by the BCA assay, and the particle/protein ratio was evaluated. Peak 1 of the SEC-purified EVs contained 1.1 ± 0.2 × 10^9^ particles/μg of protein (Fig. 2F), while the concentrate exhibited significantly lower ratios, with values of 0.01 ± 0.001 × 10^9^ particles/μg of protein. Therefore, implementing Superose 6 in the SEC purification method proved to be significantly more efficient than pretreatment with ultracentrifugation alone, resulting in at least 30-fold greater purity and an approximate recovery rate of 15 % (according to NTA of the concentrated and SEC-purified EVs – Fig. 2F). Given that objects larger than 30 nm are typically found in the interparticle volume during SEC, detecting the presence of NanoLuc in the elution volume that follows the interparticle volume emphasizes the importance of meticulous EV purification, even for *in vitro* experiments and EV characterization. This is crucial because not all delivered cargo may necessarily be loaded into EVs, which can affect the observed efficiency of pharmaceutical delivery.

### 2.2 Production of targeted engineered EVs

**Fig. 3:**
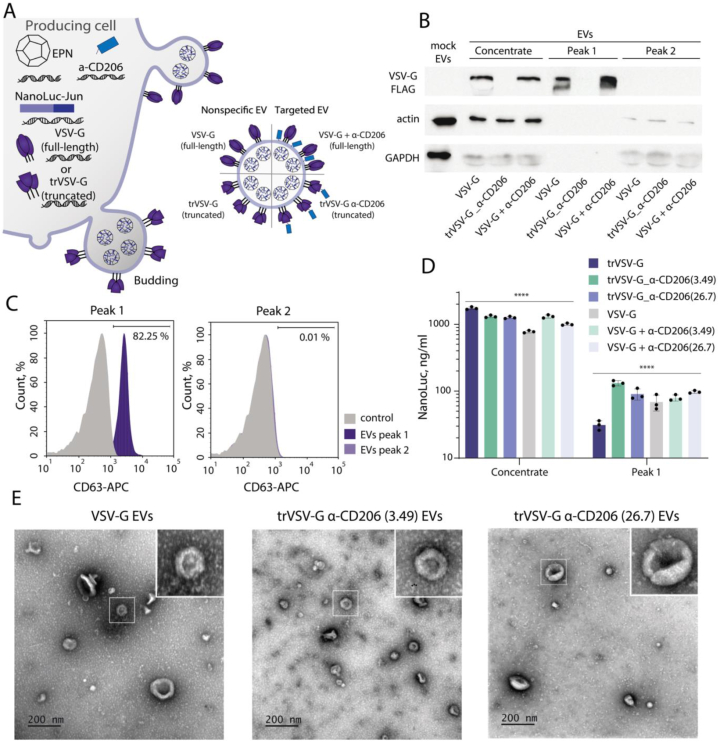
**Characterization of engineered EVs.** (**A**) Schematic illustration of the targeted engineered EV production by transfected eukaryotic cells. HEK293T cells were co-transfected with EPN, NanoLuc-Jun and VSV-G plasmids. EPN nanocages loaded with NanoLuc-Jun (recombinant luciferase) and pseudotyped with full-length or truncated VSV-G are secreted into the culture media by producer cells. An anti-CD206 nanobody was utilized to obtain targeted EVs. (**B**) Western blot analysis of the presence of VSV-G-FLAG, actin and GAPDH in SEC-purified (peak 1 and peak 2) EVs probes or media concentrates. (**C**) Flow cytometry analysis of the CD63 surface marker on SEC-purified EVs (peak 1 and peak 2) captured on the surface of CD81 magnetic beads. The grey histograms show the signals of control empty beads stained with the same antibodies. (**D**) The level of loaded NanoLuc in the samples after concentration and SEC purification. (**E**) TEM images of EVs with full-length VSV-G and truncated VSV-G fused with anti-CD206 Nbs (clone 3.49 and clone 26.7). “VSV-G” – EVs with full-length VSV-G, “trVSV-G” – EVs with truncated VSV-G, “VSV-G + α-CD206” – EVs with full-length VSV-G and the membrane-anchored anti-CD206 nanobody, “trVSV-G_α-CD206” – EVs with the truncated form of VSV-G fused with the anti-CD206 nanobody, mock EVs – natural EVs obtained from cultural media. The data are mean ± SD, n = 3, ∗∗∗∗p < 0.0001, one-way ANOVA.

For the targeted delivery of EV cargo to APCs, characterized by high surface CD206 levels, we incorporated anti-CD206 nanobody (clone 3.49 or clone 26.7) [32] onto the EV surface. Although ELISA results demonstrated that soluble recombinant Nb clone 3.49 exhibited significantly higher binding activity to recombinant human CD206 compared to clone 26.7, FACS analysis revealed that both Nbs effectively bind to activated human CD206^+^ APCs (Fig. S1). To display the required nanobody on the outer membrane of EVs and to reduce the nonspecific uptake of engineered EVs by mammalian cells, the ectodomain sequence of VSV-G was replaced with a codon-optimized 3.49/26.7 Nb sequence. To study the impact of a nanobody on EV production and loading, we compared EVs obtained after transfection with three different types of pseudotyping molecules: full-length VSV-G (“VSV-G”), full-length VSV-G in combination with the anti-CD206 nanobody fused with the transmembrane PDGFR anchor (“VSV-G + α-CD206”), and truncated VSV-G fused with the anti-CD206 nanobody (“trVSV-G_α-CD206”) (Fig. 3A). The analyzed EVs were concentrated and purified by SEC. The presence of VSV-G-FLAG was assessed via Western Blot analysis using an anti-FLAG antibody (Fig. 3B). A truncated form of VSV-G lacking the 3xFLAG tag was used as a negative control for Western Blot analysis. We have also demonstrated the absence of calnexin in SEC-purified EVs (Fig. S2). Flow cytometry analysis showed that SEC-purified engineered EVs from peak 1 expressed the specific canonical tetraspanin markers CD63 and CD81 (Fig. 3C, Fig. S3). We observed that the addition of Nb to full-length VSV-G didn't affect the EV expression and cargo loading (Fig. 3B–**D**).

The TEM images in Fig. 3E display EVs containing full-length VSV-G or trVSV-G fused with anti-CD206 Nbs. Most of the particles demonstrated a cup-shaped morphology. According to TEM, the sizes of the EVs were 99 ± 31 nm, 87 ± 41 nm and 93 ± 37 nm, for the EVs harboring full-length VSV-G, trVSV-G fused with anti-CD206 nanoantibody (clone 26.7) and trVSV-G fused with anti-CD206 nanoantibody (clone 3.49) correspondingly.

### 2.3 Targeted EV delivery into CD206^+^ cells

To enhance the precision and efficacy of engineered EV delivery to human CD206^+^ cells, we compared two different anti-CD206 nanobodies, namely, 3.49 and 26.7 [32]. We obtained three types of EVs pseudotyped with truncated VSV-G: (*i*) untargeted truncated VSV-G only, (*ii*) targeted truncated VSV-G fused with the 3.49 nanobody, or (*iii*) targeted truncated VSV-G fused with the 26.7 nanobody. We demonstrated that NanoLuc delivered by EVs decorated with truncated VSV-G was indeed internalized within recipient cells. This was confirmed using a protease protection assay, where intracellularly delivered cargo can only be digested by a proteinase in the buffer if the cell membranes are disrupted by detergents (Fig. S4). Next, we compared two protocols to generate a heterogeneous population of activated APCs containing both CD206^+^ and CD206^-^ fractions from human PBMCs: one utilizing granulocyte-macrophage colony-stimulating factor (GM-CSF) alone and the other employing a combination of GM-CSF and interleukin-4 (IL-4) (Fig. S5). Stimulation of PBMCs with GM-CSF alone resulted in a more heterogeneous population, with 17 % CD206^+^ cells, whereas stimulation with GM-CSF and IL-4 led to over half of the cells being CD206^+^. Consequently, we choose to use GM-CSF alone, as the lower proportion of CD206^+^ cells reduces the probability of EVs delivering cargo non-specifically to CD206^+^ cells.

**Fig. 4:**
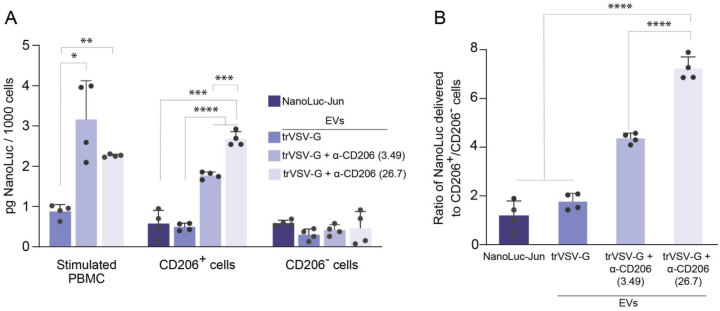
**Targeted delivery of EVs to stimulated CD206**^**+**^**antigen-presenting cells from peripheral human blood.** (**A**) The level of the delivered NanoLuc in stimulated PBMCs, and in two subpopulations after the sorting: CD206^+^ and CD206^-^. (**B**) The ratio of NanoLuc delivered to CD206^+^ versus CD206^-^ cells by different types of the EVs and soluble recombinant NanoLuc-Jun. “NanoLuc-Jun” – recombinant NanoLuc; “trVSV-G” – EVs contained truncated form of VSV-G; “trVSV-G - α-CD206 (3.49/26.7)” – EVs containing a truncated form of VSV-G fused with 3.49 or 26.7 anti-CD206 nanobody. The data are presented as mean ± SD, n = 4. ∗p < 0.05, ∗∗p < 0.01, ∗∗∗p < 0.001, ∗∗∗∗p < 0.0001, two-tailed Student's t-test.

Next, all three types of EVs loaded with NanoLuc were incubated with a heterogeneous population of GM-CSF stimulated human PBMCs for 2 h, and then, the cells were thoroughly washed to remove any uncaptured EVs, followed by staining with anti-CD206-PE antibodies. The stained cells were then sorted into CD206^+^ and CD206^-^ subpopulations. EVs decorated with both 3.49 and 26.7 anti-CD206 nanobodies were predominantly delivered to CD206^+^ cells, while no significant difference in delivery levels was observed for untargeted EVs with the truncated VSV-G or for the recombinant NanoLuc luciferase (Fig. 4). Although we did not observe any significant difference in the delivery efficiency between the 3.49 and 26.7 anti-CD206 nanobodies for the total pool of stimulated PBMCs, further analysis of separate CD206^+^ and CD206^-^ subpopulations revealed that the delivery of EVs with the 26.7 anti-CD206 nanobody was more specific, than that with the 3.49 anti-CD206 nanobody.

## 3 Discussion

Professional APCs mainly include Mϕs and DCs [33]. They are capable of capturing, processing, and presenting antigens to T cells, significantly modulating immune responses. Therefore, therapeutic delivery of target pharmaceuticals to these cells can be implemented for the treatment of oncological [34,35], viral [36], and autoimmune diseases [[37], [38], [39], [40]]. Genetically engineered EVs can serve as a fine-tunable and flexible solution for the efficient delivery of protein cargoes to eukaryotic cells. In this study, our objective was to establish a robust method for targeting functional EVs to APCs. According to preclinical studies, EVs are predominantly taken up by Mϕs and DCs [41]; however, some of them are taken up by other cells. The use of VSV-G-based EVs to an even greater extent is followed by their nonspecific uptake by a variety of mammalian cells expressing common low-density lipoprotein receptor (LDL-R) on the surface [14,[42], [43], [44]]. We demonstrated the functionality of the truncated VSV-G fused with two different nanobodies to ensure EV assembly. Furthermore, we optimized the purification method to harvest functional and purified engineered EVs.

CD206 (also known as the mannose receptor) was chosen as a target due to its increased expression in mature APCs [28,29,45,46]. In our study, we compared two llama nanobodies against CD206, namely, 3.49 and 26.7 [32]. These clones exhibit different affinities for the mannose receptor across different mammalian species (mouse, rat, rabbit, and human). Despite the high structural homology between mannose receptors of different species, their amino acid sequences still display certain variability. Both clones demonstrated efficient delivery of luciferase cargo to human CD206^+^ cells, with clone 26.7 showing slightly higher uptake by CD206^+^ antigen-presenting cells. We further demonstrated that utilizing the monoclonal anti-CD206 nanobody fused with a truncated form of VSV-G, characterized by a weaker interaction with LDL-R [43,47], significantly improved the specificity of preferable EV uptake by CD206^+^ cells. This breakthrough not only holds promise for the targeted modulation of immune responses via CD206 targeting but also presents a novel avenue for drug delivery to tumors harboring specific antigens. Given the ability of the engineered EVs to deliver proteins directly into the cytoplasm of target cells while bypassing lysosomal degradation, our future studies will focus on exploring this platform's potential for functional antigen transfer. Specifically, we plan to assess its capacity to facilitate the presentation of delivered peptides in complex with MHC class I molecules, aiming to deliberately activate CD8^+^ T cells for a specific immune response.

## 4 Limitations of the study

In this study, we optimized the platform for the production and purification of targeted EVs engineered for the specific delivery of a cargo protein to APCs. To assess the effectiveness of protein delivery to such cells, we utilized the model cargo protein NanoLuc. Further investigation is required to explore the engineered EVs' ability to modulate the immune response via therapeutic delivery to APCs in the context of other immunogenic antigens. The second challenge of using engineered EVs for clinical applications lies in the substantial costs linked to their production, mainly due to the low yield of purified preparations. Lastly, it would be of great interest to conduct *in vivo* studies to evaluate the immunological outcomes of EV applications.

## Materials and methods

### Cell lines and primary cells

HEK293T cells were obtained from Russian Cell Culture Collection (RCCC, Institute of cytology of the Russian Academy of Sciences). Authentication of HEK293T was performed using Short Tandem Repeat (STR) analysis at the beginning of the experiments with EVs production, prior to HEK293T cryopreservation, and after the first week of cell culture. HEK293T cells were cultured in DMEM medium (Gibco, USA) supplemented with 2 mM GlutaMax (Gibco, USA), 10 % exosome-depleted fetal bovine serum (FBS) (Gibco, USA), and 100 U/ml penicillin-streptomycin (Gibco, USA). The cells were maintained at 5 % CO_2_ and 37 °C in a humidified incubator. Routine mycoplasma testing was performed on HEK293T cells using MycoReport Kit (Evrogen, Russia) to confirm the absence of contamination.

Stimulated DCs and Mϕs were obtained from human peripheral blood mononuclear cells (PBMCs). PBMCs were obtained from healthy donors. The study protocol was approved by the independent Ethics Committee of Central Clinical Hospital of the Russian Academy of Sciences (protocol #143). All donors provided written informed consent for enrollment, followed by a discussion of the study with the investigators. PBMCs were isolated by Ficoll-Paque density gradient centrifugation. Subsequently, the isolated PBMCs (10-15 x 10^6^ cells per T25 flask) were incubated in complete RPMI medium supplemented with 10 % FBS. Following a 4-h incubation period at 37 °C in a humidified incubator with 5 % CO_2_, the nonadherent cells were removed. Then, 100 ng/ml of human granulocyte-macrophage colony-stimulating factor (hGM-CSF) (Gibco, USA) alone or combination of 100 ng/ml hGM-CSF and 50 ng/ml of Interleukin-4 (IL-4) (SciStoreLaboratory, LTD, Russia) were added to the adherent precursors of DCs and Mϕs. The differentiation of mononuclear cells into DCs and Mϕs was induced by cultivating them in the presence of GM-CSF/GM-CSF plus IL-4 for six days, and the medium was changed every two days.

### Plasmids

pCMV-EPN-Fos-C (Addgene ID: 167306) and pCMV-NanoLuc-Jun (Addgene ID: 167308) have been previously described and are accessible through the Addgene plasmid repository [14]. The sequence corresponding to a truncated form of VSV-G was amplified from the full-length VSV-G (AddgeneID: 138479). Sequences encoding llama nanobodies (Nbs) (clones 3.49 and 26.7) [32] were synthesized and cloned at the 5′-end of the truncated VSV-G into the pCMV-VSV-G_truncated construct, and a 3xFLAG epitope was added to the protein C-terminus. To obtain recombinant Nbs, the sequences of clones 3.49 and 26.7 were cloned into the pET32 expression vector, His-tag and 3xFLAG epitopes were added.

### Recombinant a-CD206 Nbs expression

Recombinant anti-CD206 Nbs clones 3.49 and 26.7 were produced using a prokaryotic expression system in *Escherichia coli BL21(DE3)* cells. Overnight cultures were inoculated into fresh 2 × YT medium at a 1:100 dilution and grown at 37 °C with shaking until the optical density at 600 nm (OD₆₀₀) reached 0.4–0.6. Protein expression was induced by adding 1 mM isopropyl β-D-1-thiogalactopyranoside (IPTG), and the cultures were incubated at 28 °C with aeration for 16 h. Cells were harvested by centrifugation at 3500×*g* for 10 min at 4 °C. The cell pellet was resuspended in lysis buffer containing 50 mM Tris-HCl (pH 8.0), 150 mM NaCl, 1 mM phenylmethylsulfonyl fluoride (PMSF), and 0.2 mg/mL lysozyme. The suspension was incubated at room temperature until it became viscous, indicating cell lysis. The cell lysate was further disrupted using ultrasonic homogenization. The resulting solution was centrifuged at 20,000×*g* for 10 min at 4 °C to remove cell debris. The supernatant was filtered through a 0.45 μm membrane filter and loaded onto a Ni-NTA affinity column (Qiagen, Germany) pre-equilibrated with binding buffer (50 mM Tris-HCl, pH 8.0; 150 mM NaCl). Impurities were removed by sequentially washing the column with binding buffer and wash buffer containing 20 mM imidazole. The recombinant Nbs were eluted using elution buffer (50 mM Tris-HCl (pH 8.0), 150 mM NaCl, and 350 mM imidazole). Further purification of the nanobodies was carried out using SEC with a Superdex 75 column (Cytiva, USA) following standard protocols.

### EV preparations

HEK293T cells were seeded into T25 flasks at a cell density of 0.4 × 10^6^ cells/ml to a final volume of 5 ml. The next day, cells that reached 80–90 % confluency were transfected with the following plasmid constructs: 4.5 μg pCMV-EPN-Fos-C, 2.5 μg pCMV-NanoLuc-Jun, and 0.5 μg pCMV-VSVG or pCMV-VSV-G_truncated or pCMV-VSV-G_truncated_3.49 or pCMV-VSV-G_truncated_26.7. Transfection was carried out using the Lipofectamine 3000 transfection reagent (Invitrogen, USA). Following a 6-h incubation, the culture medium was replaced with Advanced DMEM supplemented with 5 % exosome-depleted FBS (Gibco, USA). EVs were harvested after 48 h.

The culture media containing EVs were subjected to differential low-speed centrifugation steps to eliminate cell debris and microparticles (300×*g* for 10 min, 4000×*g* for 15 min) and then filtered through a 0.45 μm filter device (GE Healthcare, UK). The filtered medium containing EVs was concentrated using an Amicon Ultra15 100 kDa Filter Unit (Millipore, Ireland) and subjected to three washes with PBS (Gibco, USA).

The concentrated EVs were loaded on a Superose 6 10/300 GL column (Cytiva, USA) equilibrated with PBS, and 500 μl fractions were collected starting at an elution volume of 5 ml. The fractions were analyzed using a NanoGlo Luciferase Assay (Promega, USA) in accordance with the manufacturer's protocol. Total protein content in the fractions was determined through a Pierce BCA Protein assay (Thermo Scientific, USA).

To ensure that the investigation focused on EVs and not adventitious nanoparticles, we utilized a blank EV sample, which consisted of culture medium incubated without cells in the same flasks under the same conditions as the engineered EV preparation process, and subsequently subjected to all purification steps.

### Nanoparticle tracking analysis (NTA)

The particle size and concentration were measured using a NanoSight LM10 HS-BF instrument (NanoSight Ltd, UK) in the following configuration: 405 nm 65 mW laser unit with passive temperature readout, high-sensitivity camera of EMCCD type, and NanoSight 2.3 build 0033 software. For each sample, first, an appropriate sample dilution (10–3000) with particle-free PBS was selected to reach the optimal concentration of around 1.5 × 10^8^ particles/ml. Camera and processing setting were used as previously optimized for EVs [48,49]: Camera Shutter = 850, Camera Gain = 450, Lower Threshold = 910, Higher Threshold = 10920, Detection Threshold = 9 Multi, Min Expected Size = 30 nm. For samples of conditioned medium with high background which resulted from scattering of protein molecules and riboflavin fluorescence, the Lower and Higher Thresholds were shifted to 1885 and 11895, respectively. Fourteen to twenty-one 60 s videos were recorded for each sample to reach several thousand tracks in total. Based on the data from individual runs, average particle size distribution and total particle concentration corrected by dilution factor were obtained.

### Western Blot

Western Blot analysis was performed as previously described [14] with minor modifications. For the concentrates, 20 μg of EVs (total protein) were loaded, while 22.5 μl of sample (maximum well capacity) were used for the SEC eluates. The samples were mixed with the Laemmli buffer (125 mM Tris-HCl (pH = 6.8), 4 % (w/v) SDS, 10 % (w/v) 2-mercaptoethanol, 20 % (w/v) glycerol, 0.004 % (w/v) bromphenol blue) at a ratio of 1:4 (v/v) followed by heating for 5 min at 95 °C. The samples were loaded on 12 % polyacrylamide gel, and after the electrophoresis, the proteins were transferred to a 0.45 μm nitrocellulose membrane (Amersham, Germany). The membrane was blocked with 5 % (w/v) milk in PBS supplemented with 0.05 % (v/v) Tween-20 (Helicon, Russia) and incubated with monoclonal HRP-conjugated anti-FLAG M2 antibodies (Sigma-Aldrich Cat# A8592, RRID:AB_439702), anti-actin-HRP antibodies (Sigma-Aldrich Cat# A3854, RRID:AB_262011), anti-GAPDH mouse antibodies (ServiceBio Cat# GB15002, RRID:AB_3096308) followed by anti-mouse secondary antibodies (Abcam Cat# ab97240, RRID:AB_10695944), or with anti-calnexin mouse antibodies (Abcam Cat# ab31290, RRID:AB_868628), followed by anti-mouse secondary antibodies. Clarity Western ECL Blotting Substrate (Bio-Rad Laboratories Inc., USA) was used for chemiluminescence detection of proteins.

### 7.7 ELISA

96-well MaxiSorp plates (Nunc, Denmark) were coated with 50 μL of recombinant human CD206 protein (Antibody System Cat#EHD55201) at a concentration of 4 μg/mL in carbonate buffer overnight. Plates were then washed with 250 μL of PBST and blocked with 250 μL of 2 % nonfat dry milk in PBS for 1 h at 37 °C. The purified Nbs were diluted in conjugate buffer (PBST with 0.5 % nonfat dry milk). 50 μL of the purified Nbs were added to each well and incubated for 1 h at 37 °C. Then the plates were washed 3 times with wash buffer. Anti-FLAG M2-Peroxidase (HRP)-conjugated antibodies (Sigma-Aldrich Cat# A8592, RRID:AB_439702) were diluted 1:20000 in conjugate buffer, and 50 μL was added to each well. After 1 h at 37 °C, the plates were washed 5 times with wash buffer and 50 μL of TMB substrate was added. After 10 min at room temperature, the reaction was stopped with 50 μL of 10 % phosphate acid, and the OD_450_ was read on a VarioScan plate reader.

### Flow cytometry analysis

To characterize the pattern of surface antigens, EVs were captured on magnetic anti-CD81 beads (Exosome-Human CD81 Flow Detection Reagent, Invitrogen) through an overnight incubation at +4 °C with constant gentle rotation, followed by a thorough wash with assay buffer (PBS, 0.1 % BSA). EVs attached to anti-CD81 beads were stained with anti-CD63 antibodies (BioLegend Cat# 353007, RRID:AB_10916393) and analyzed by flow cytometry on a NovoCyte 2060 (ACEA Biosciences, USA). The data were analyzed with NovoExpress Software (ACEA Biosciences, USA).

To stain APCs with recombinant anti-CD206 Nbs, GM-CSF and IL-4 stimulated cells were washed twice with phosphate-buffered saline (PBS) and resuspended in Zombie Violet solution. Cells then were washed and resuspended in a solution containing 30–150 μg/mL of recombinant anti-CD206-FLAG Nb (clone 3.49 or 26.7). The cells were incubated at 4 °C with gentle agitation for 1 h. After incubation, the cells were washed twice with PBS and stained with anti-FLAG-APC antibody (BioLegend Cat# 637308, RRID:AB_2561497). For control staining, anti-CD206-PE antibody (BioLegend Cat# 321106, RRID:AB_571911) was used. HEK293T cells were stained as a negative control.

### Transmission electron microscopy (TEM)

Carbon-coated TEM grids (Ted Pella, USA) were treated using a Emitech K100X glow discharge device (Quorum Technologies Ltd., UK) at a current of 25 mA for 45 s. This treatment turned the carbon surface hydrophilic and increased EV adsorption. The suspension of EVs was deposited onto the grid for 2–3 min, and the liquid was blotted using filter paper (OdiChem, Russia). The grids were quickly transferred onto the drops of 1 % uranyl acetate, stained for 1–2 min, blotted and dried. Images were obtained using a JEM-1400 (Jeol, Japan) transmission electron microscope equipped with a Rio-9 camera (Gatan Inc., USA). The magnification ranged from 30,000 × to 150,000 × , and the acceleration voltage was 120 kV. The sizes of the EVs were measured using ScanEV [50].

### Analysis of the efficiency and specificity of the delivery by targeted EVs

Concentrates or SEC-purified EVs containing NanoLuc were standardized according to the luciferase signal and added to a heterogeneous population of one million APCs (DCs and Mϕs) stimulated from human peripheral blood. Recombinant soluble NanoLuc was used as a control. Following a 2-h incubation at 37 °C with 5 % CO_2_, the cells were washed with PBS (Gibco, USA), subsequently incubated in PBS with 50 μg/mL Proteinase K (Evrogen, Russia) for 15 min at 37 °C with continuous rotation. After incubation, the cells were washed three times with PBS (Gibco, USA) and stained with an anti-human anti-CD206-PE antibody (BioLegend Cat# 321105 (also 321106), RRID:AB_571910). All centrifugation steps were performed at 300×*g* for 10 min. The CD206-positive and CD206-negative cell subsets were separated using Fluorescence-Activated Cell Sorting (FACS) on a Sony SH800 cell sorter (Sony, Germany). A total of 10,000 cells were used to assess the quantity of the delivered NanoLuc with the Nano-Glo Luciferase Assay. The signal was detected using a Varioskan plate reader (Thermo Scientific, USA) at 460 nm. Each biological repeat for luciferase assay was measured at least in triplicate (technical repeat).

### Proteinase K protection assay

Jurkat cells were incubated with EV samples for 2 h at 37 °C, followed by washing with PBS. The cells were then incubated in PBS, either with or without 1 % Triton X-100 and 50 μg/mL Proteinase K (Evrogen, Russia), for 15 min at 37 °C with continuous rotation. After incubation, 10 μL of the cell suspension was mixed with 10 μL of Nano-Glo Luciferase Assay Buffer containing Nano-Glo substrate (Promega, USA). The remaining NanoLuc activity was immediately measured using a Varioskan plate reader (Thermo Scientific, USA) at 460 nm.

### Statistical analysis

Statistical analysis was performed with GraphPad Prism 9. The numerical data is provided as mean ± standard deviation. The significance of differences was assessed using the two-tailed Student's t-test or one-way ANOVA. P-values <0.05 were considered to be significant.

## CRediT authorship contribution statement

**Leyla A. Ovchinnikova:** Writing – review & editing, Writing – original draft, Methodology, Investigation, Formal analysis, Data curation, Conceptualization. **Daria Y. Tanygina:** Writing – original draft, Investigation. **Samir S. Dzhelad:** Investigation. **Evgeniy G. Evtushenko:** Visualization, Methodology, Investigation. **Dmitriy V. Bagrov:** Visualization, Methodology, Investigation. **Alexander G. Gabibov:** Writing – review & editing, Supervision, Resources, Conceptualization. **Yakov A. Lomakin:** Writing – review & editing, Writing – original draft, Supervision, Project administration, Formal analysis, Conceptualization.

## Ethics declaration

The use of human specimens was reviewed and approved by the independent Ethics Committee of Central Clinical Hospital of the Russian Academy of Sciences (protocol #143 from June 26, 2020) in conformity with the ethical principles of the Helsinki Declaration and with Patients’ consent and approval.

## Data availability statement

The authors confirm that the data supporting the findings of this study are available within the article and its supplementary materials.

## Declaration of competing interest

The authors declare that they have no known competing financial interests or personal relationships that could have appeared to influence the work reported in this paper.
