# Supplementary material for: Targeted macrophage mannose receptor (CD206)-specific protein delivery via engineered extracellular vesicles
Source: Heliyon. 2024 Dec 6;10(24):e40940. doi: 10.1016/j.heliyon.2024.e40940 (PMC11697562; doi:10.1016/j.heliyon.2024.e40940)
Supplement: Multimedia component 1 [file mmc1.docx]

**Supplemental Material**

**Targeted macrophage mannose receptor (CD206)-specific protein delivery via engineered extracellular vesicles**

**by Ovchinnikova LA et al.**


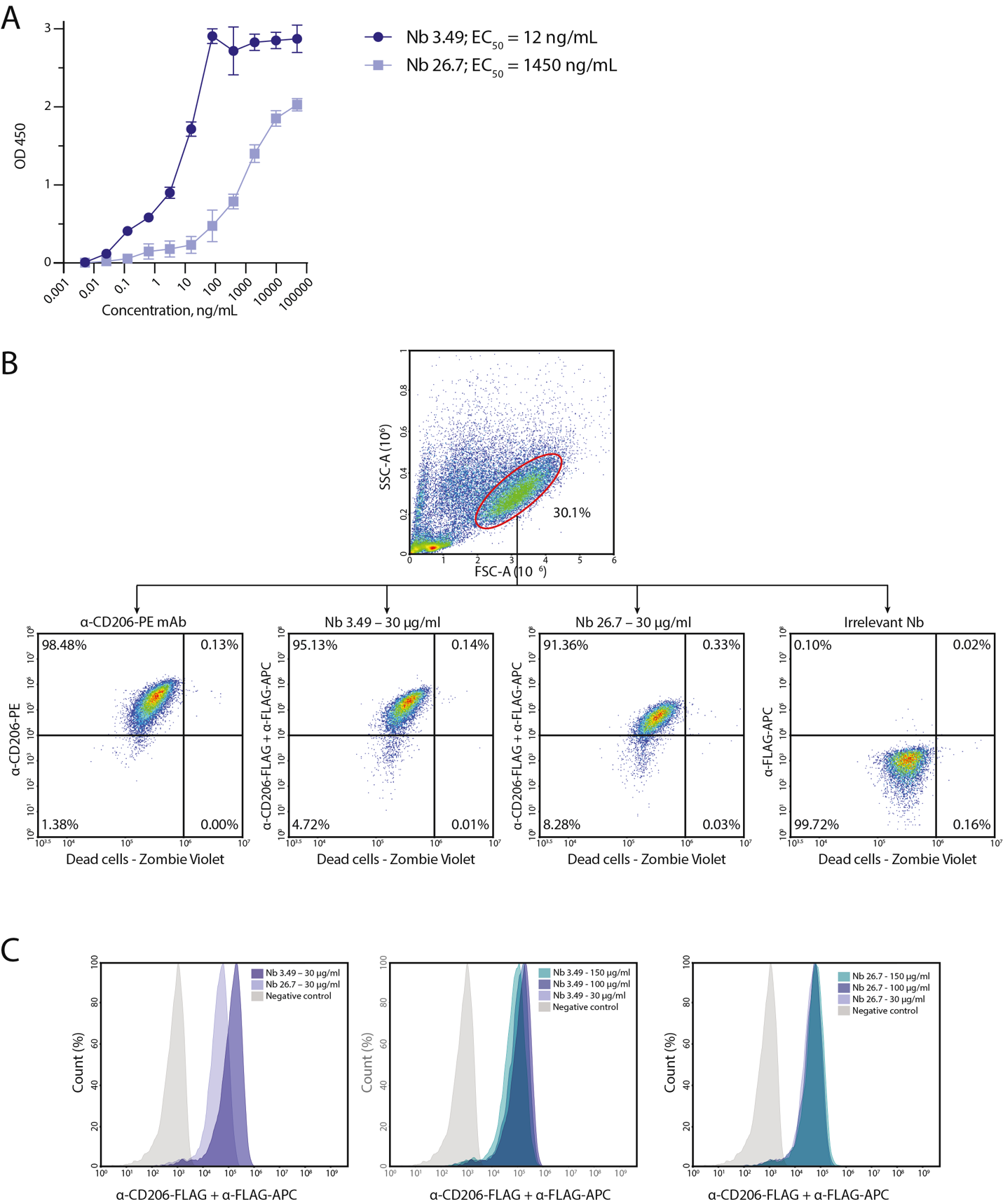


**Figure S1. Binding analysis of recombinant anti-CD206 nanobody (Nb) clones 3.49 and 26.7 to CD206.**

(**A**) ELISA demonstrating the binding affinity of recombinant anti-CD206 Nbs 3.49 and 26.7 to human recombinant CD206. (**B**) Representative density plots and (**C**) histograms illustrating flow cytometry analysis of APC stained with the recombinant anti-CD206 Nbs 3.49 and 26.7. Differentiation of APC (DCs and Mϕs) from human peripheral blood mononuclear cells was stimulated using IL-4 and GM-CSF for 6 days. Cell binding with the recombinant anti-CD206 Nbs fused with FLAG-tag was visualized with a fluorescent secondary antibody, anti-FLAG-APC. A commercially available anti-CD206-PE antibody was used as a positive control. Negative controls included cells stained with irrelevant Nb or CD206-negative cells (HEK293T).


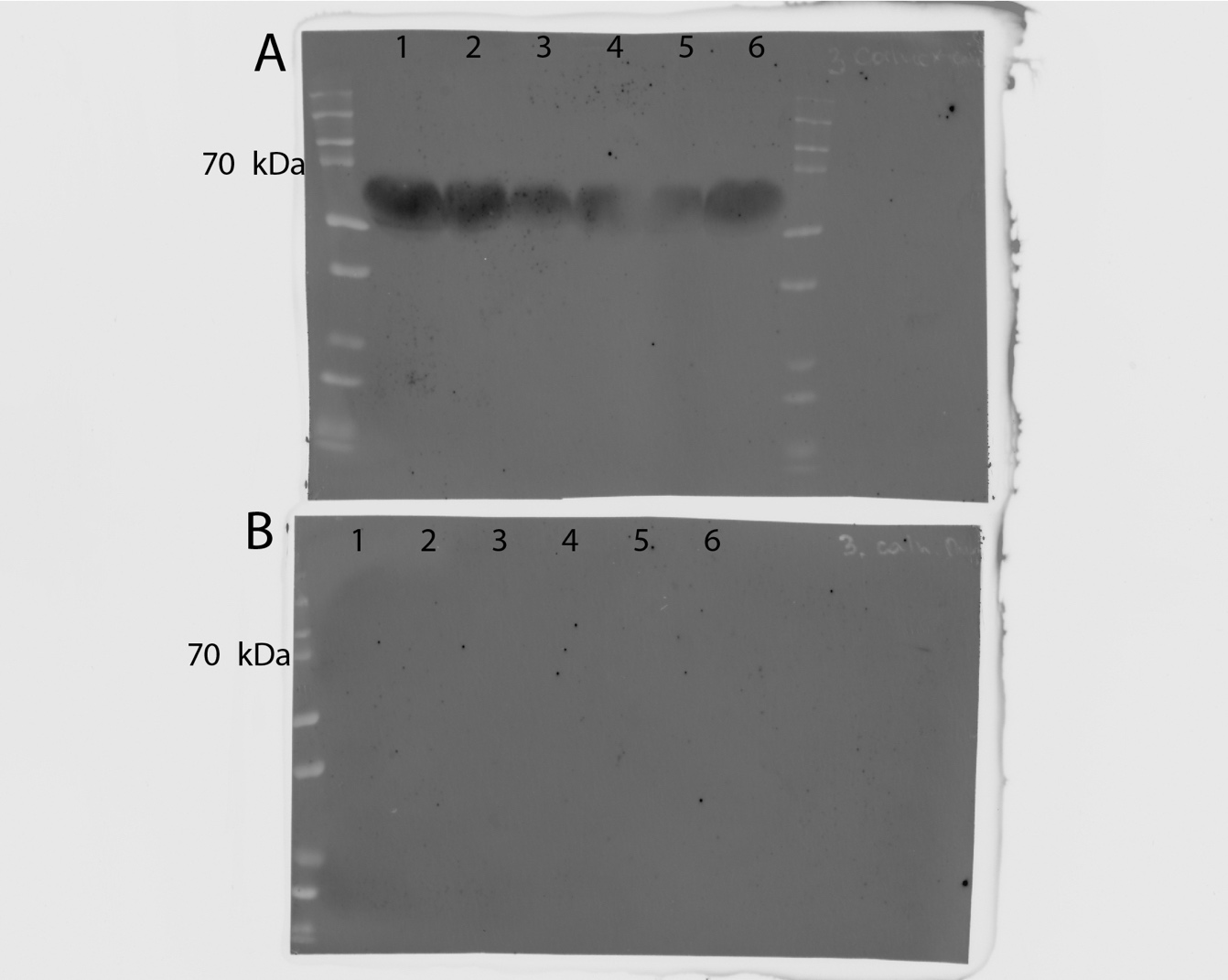


**Figure S****2.** Western blot analysis of the presence of calnexin in media concentrates (**A**) and SEC-purified (peak 1) EVs samples (**B**). “1” – EVs contained truncated form of VSV-G, “2” – EVs contained truncated form of VSV-G fused with 3.49 anti-CD206 nanobody, “3” – EVs contained truncated form of VSV-G fused with 26.7 anti-CD206 nanobody, “4” – EVs contained full-length form of VSV-G, “5” – EVs contained truncated form of VSV-G and the membrane-anchored anti-CD206 nanobody (clone 3.49), “6” – EVs contained truncated form of VSV-G and the membrane-anchored anti-CD206 nanobody (clone 26.7).


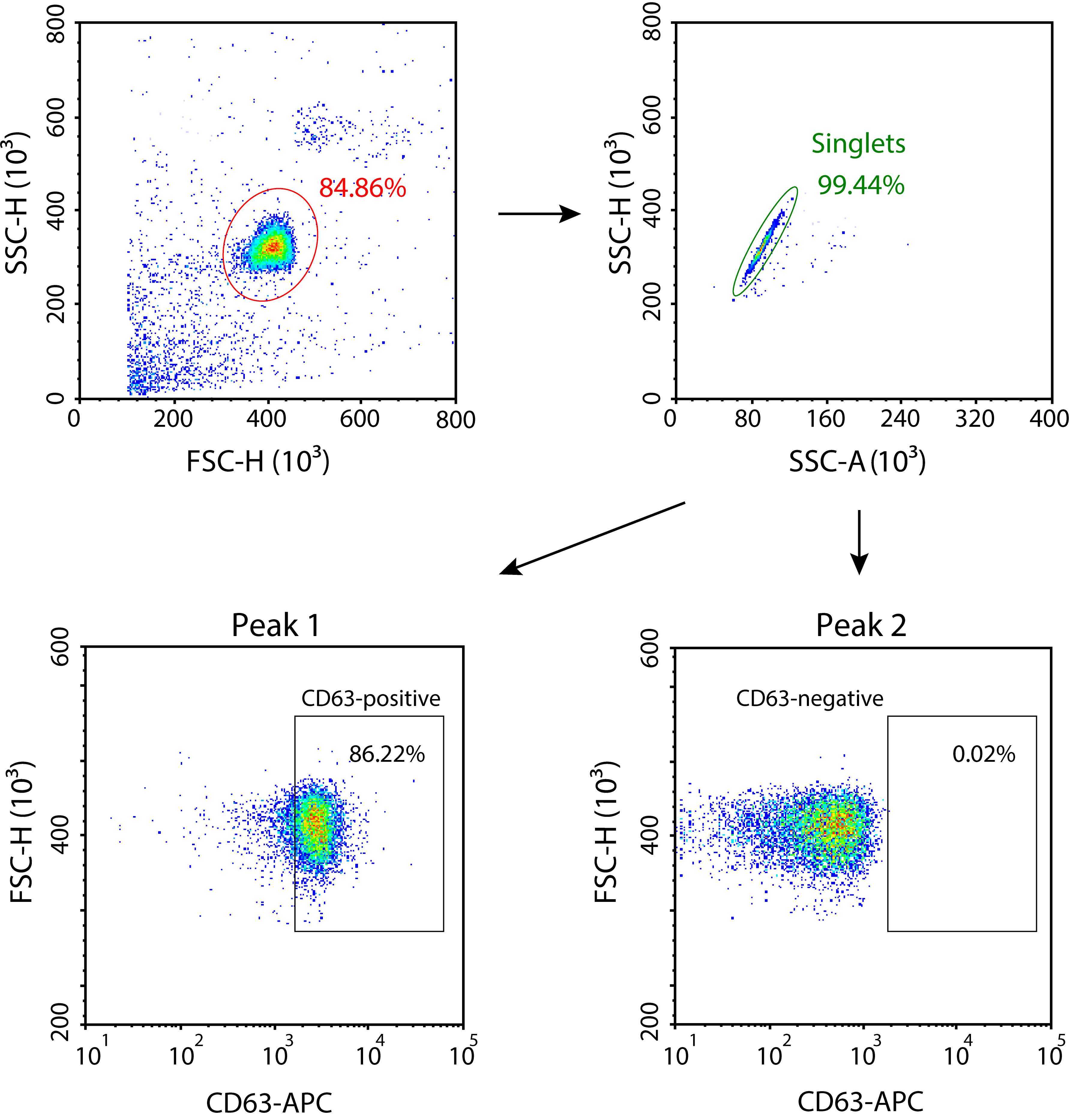


**Figure S3. Gating strategy for flow cytometry analysis of EVs, captured on anti-CD81 magnetic beads.** Beads with captured EVs were initially gated to select the total bead population while excluding debris. Subsequently, single non-aggregated vesicles-bead complexes (singlets) were identified. SEC-purified EVs (Peak 1 and Peak 2) were then analyzed for the presence of CD63.

) ELISA analysis of the binding affinity between recombinant α-CD206 Nbs 3.49 and 26.7 with human recombinant human CD206.

(B) Representative density plots and (C) histograms illustrating flow cytometry analysis of APC staining with the recombinant α-CD206 Nbs 3.49 and 26.7.

APC (DC and MP) differentiation from human peripheral blood MNCs was stimulated by using IL-4 and GM-CSF for 7 days.

Cell binding with the recombinant α-CD206 Nbs fused with FLAG-tag was visualized with a fluorescent secondary antibody α-FLAG-APC, or a commercially

available antibody, α-CD206-PE. Negative controls included cells stained with irrelevant Nb or CD206-negative cells (HEK293T).


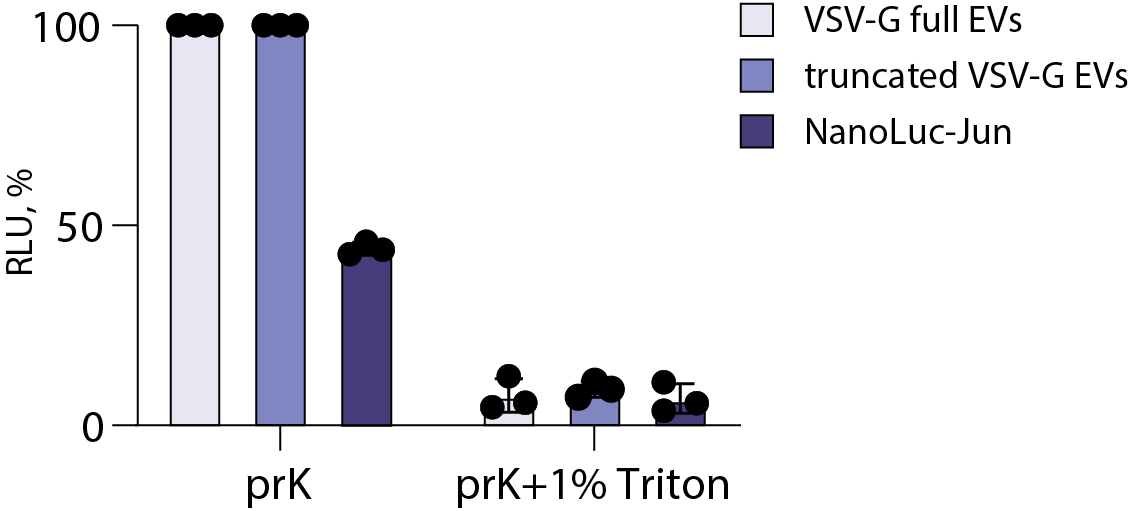


**Figure S4. Protease protection assay of NanoLuc delivered to model cell line.** Jurkat cells were treated with Proteinase K alone (prK) or with Proteinase K in combination with 1% Triton X-100 (prK+1%Triton), then washed and analyzed using the Nano-Glo luciferase assay. The level of NanoLuc delivered inside the cells (cells incubated with VSV-G full EVs and treated with Proteinase K alone) was set to 100%. Each dot is an independent replicate and represents the mean of 3 technical replicates. Error bars represent standard deviation (SD). RLU – relative light units.


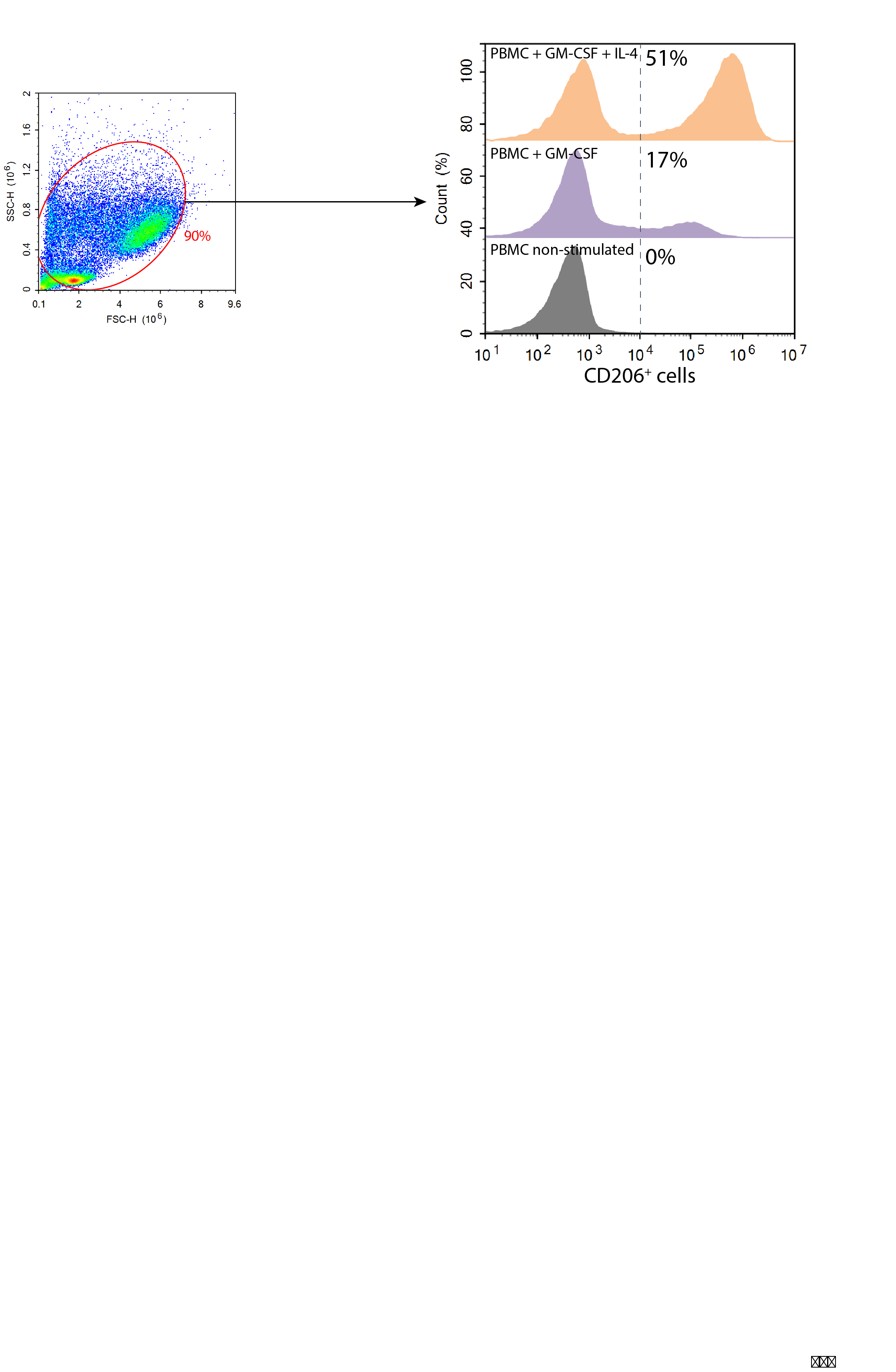


**Figure S5. Stimulation of Antigen-Presenting Cells from Human PBMCs.**
Gating strategy and histograms illustrating the flow cytometry analysis of cells stained with anti-CD206-PE antibodies are shown. To generate a heterogeneous population of antigen-presenting cells (both CD206-positive and CD206-negative), human PBMCs were incubated for 6 days under two different conditions: with GM-CSF and IL-4 (orange) or with GM-CSF alone (violet). For comparison, fresh non-stimulated PBMCs are shown in grey.
